# Editorial: Women in bioinformatics

**DOI:** 10.3389/fbinf.2024.1499514

**Published:** 2024-10-08

**Authors:** Irma Martínez-Flores, Constanza Cárdenas Carvajal, Viviana Monje-Galvan

**Affiliations:** ^1^ Centro de Ciencias Genómicas, Universidad Nacional Autónoma de México, Cuernavaca, Morelos, Mexico; ^2^ Nucleo Biotecnologia Curauma, Pontificia Universidad Católica de Valparaíso, Valparaíso, Chile; ^3^ Department of Chemical and Biological Engineering, State University of New York at Buffalo, New York, NY, United States

**Keywords:** women in bioinformatics, gender equality, computational tools, innovation approaches, inclusive environment

There has been much progress towards gender equality over the past decade, yet women continue to face significant obstacles in their professional trajectories. We must continue to advocate for our role in science as participants, leaders, and innovators. To dismantle stereotypes and transform social perceptions, we must actively promote gender equality within existing structures with a historical bias in favor of men and encourage young girls and women to explore careers in STEM, including bioinformatics.

Bioinformatics, a discipline that integrates diverse areas such as biology, computer science, and statistics, has advanced rapidly, driving groundbreaking discoveries in genomics, proteomics, metabolomics, and beyond. In this multidisciplinary field, women have made important contributions that have not been recognized or disseminated as they deserve, leading to an underrepresentation of female participation. This gender gap hinders individual careers and deprives the scientific community of diverse perspectives that are crucial to solving complex biological problems.

Numerous women in science have overcome challenges in their pursuit of professional excellence. It is up to all of us to create platforms that celebrate, empower, and support their quest for professional achievements. The Research Topic we present here honors outstanding contributions of women in bioinformatics and underscores the need to continue to amplify our voices and highlight our research to inspire and shape future generations of scientists.

Through five featured articles, we celebrate the diverse perspectives and innovative research led by women in Bioinformatics and related fields. Soto et al. presents a study that provides a deep understanding of how the cellular Prion protein (PrPC) and Doppel protein interact with membrane surfaces at the molecular-level. Her study highlights the role of specific protein-lipid interactions and conformational constraints in shaping their binding modes. Dr. Soto emphasizes the implications for prion disease mechanisms through advanced molecular dynamics simulations and comprehensive analysis to investigate this complex biological phenomenon.

Ogunnupebi et al. showcase an in silico exploration to identify innovative insecticidal compounds that offer more effective and sustainable control methods to inhibit trehalase, an enzyme vital to mosquito survival. Dr Ajani and Adebiy use computer-assisted drug discovery methods to streamline the evaluation of potential pharmacological actions of chemical compounds before they are manufactured.

Gutierrez-Diaz et al. explores the role of small RNA fragments derived from non-coding RNAs (sfd-RNAs) in genetic regulation during viral infections. Her studyemphasizes the importance of utilizing a methodology that combines 16 computational strategies, including aligners and normalization methods, to ensure a reliable protocol to identify sfd-RNAs. Her team successfully identified differentially expressed miRNAs and sfd-RNAs that are crucial for dengue virus infection, demonstrating the utility and application of their proposed methodology.

Li et al. research focuses on the development of MimoTree, a depth-first search (DFS)-based algorithm, to identify antibody-binding epitopes from mimotope datasets (short peptides that mimic the structure of epitopes and bind to antibodies). The algorithm can detect conformational and linear epitopes and, when combined with other available methods, form an ensemble approach to enhance the understanding of antigen-antibody binding interactions. Her findings can aid in vaccine development and facilitate the rapid development of more specific and sensitive diagnostic immunoassays.

Finally, the mini-review by Gondal et al. provides a vital resource for researchers in the field of single-cell transcriptomics. It emphasizes the potential of single-cell RNA sequencing (scRNA-seq) to uncover biological complexities. This review offers an extensive overview of single-cell transcriptomics databases, examining their applications and limitations in scRNA-seq.

Frontiers in Bioinformatics is proactive in creating a more inclusive environment for women in science. The contributions to this Research Topic are a clear testament to women’s transformative resilience to bioinformatics. Their expertise ranges from developing single-cell databases, to tools for studying small RNAs, protein design, molecular modeling, and discovering functional regions in biologically important molecules. By celebrating the achievements of these researchers, we reaffirm our commitment to inclusivity and equality. We are committed to strengthening the spaces that amplify women’s voices in bioinformatics and other STEM fields. We invite all researchers to join in this effort to make women’s voices in bioinformatics visible. To promote a diverse and equitable workforce that drives scientific discovery enriched by unique experiences and perspectives that enable us to tackle global challenges more effectively.

Together, we can initiate significant change to uplift women in our field, foster innovation, and motivate future generations to dream big and pursue their passions in the exciting world of bioinformatics.

